# The acceptability and validity of AI-generated psycholinguistic stimuli

**DOI:** 10.1016/j.heliyon.2025.e42083

**Published:** 2025-01-17

**Authors:** Alaa Alzahrani

**Affiliations:** King Saud University, Riyadh, Saudi Arabia

**Keywords:** AI-Generated stimuli, Psycholinguistic stimuli, ChatGPT, AI for research, Auditory stimuli, Sentence stimuli

## Abstract

Sentence stimuli pervade psycholinguistics research. Yet, limited attention has been paid to the automatic construction of sentence stimuli. Given their linguistic capabilities, this study investigated the efficacy of ChatGPT in generating sentence stimuli and AI tools in producing auditory sentence stimuli. In three psycholinguistic experiments, this study examined the acceptability and validity of AI-formulated auditory sentences and written sentences in one of the two languages: English and Arabic. In Experiment 1 and 3, participants gave English AI-generated stimuli similar to or higher acceptability ratings than human-composed stimuli. In Experiment 2, Arabic AI-generated stimuli received lower acceptability ratings than their human-composed counterparts. The validity of AI-developed stimuli relied on the study design, with only Experiment 2 demonstrating the target psycholinguistic effect. These results highlight the promising role of AI as a stimuli developer, which could facilitate psycholinguistic research and increase its diversity. Implications for psycholinguistic research were discussed.

## 1 Introduction

Harnessing generative AI for research purposes has garnered widespread attention since the introduction of ChatGPT at the end of 2022 [[1], [2], [3]]. In second language and bilingual research, most of the applications of AI have been focused on data analysis [[4], [5], [6], [7], [8]] and generating norming data [9,10]. A far less examined application of AI is in stimuli development [11], possibly due to the challenges involved in sentence stimuli generation, such as syntactic complexity and context sensitivity. In the psycholinguistic literature, several methods have been proposed for the generation of word stimuli [12,13]. However, the automatic formulation of sentence stimuli remains underexplored despite the wide adoption of sentence items in the field [11]. Creating sentence stimuli for psycholinguistic studies is not an easy task. Utilizing the text generation capabilities of AI could facilitate the process of conducting psycholinguistic research.

In response to these gaps, this study aims to investigate the acceptability and validity of two types of AI-generated sentence stimuli (auditory, written) across three psycholinguistic experiments conducted in one of two languages (English, Arabic). Acceptability in the current paper refers to the extent to which AI-generated stimuli are perceived as natural as human-generated stimuli. Validity here refers to the degree to which studies with AI-generated stimuli could replicate findings from studies with human-generated stimuli (For more detailed definitions, see the literature review). The current study is one of the early studies examining AI-generated auditory and written sentence stimuli across two languages (English, Arabic) in the psycholinguistic field. To increase the generalizability of our findings, we investigated two typologically distinct languages (English, Arabic) which vary in many aspects, including their writing system (Latin vs. Abjad), morphological system (word parts are attached linearly vs. non-linearly), word order preferences (subject-verb-object vs. verb-subject-object), among others [[14], [15], [16]]. Results would reveal the extent to which AI-generated stimuli are acceptable and valid in the context of psycholinguistic research, providing practical implications for the field.

### 1.1 Literature review

#### 1.1.1 Types of psycholinguistic stimuli

Two primary linguistic stimuli formats are used in psycholinguistic research: auditory and visual. The auditory format might present the auditory form of a word [17], a sentence [18], or a short text [19] carrying the target feature. Visual stimuli may consist of written language (a word, sentence(s), a text) [20] or pictures representing a target object/event [21]. All types of stimuli are controlled in some way to allow a more robust investigation of the effect of interest. Crucially, auditory sentences are typically manually modified using specialized software (e.g., Praat, Audacity) to adjust the presence and duration of pauses [22,23]. Additionally, the voice actor may regulate the speech rate by articulating a specific number of syllables per second [18]. The current study examined AI-generated auditory and written sentence stimuli due to their frequent use across different psycholinguistic designs [24].

#### 1.1.2 Challenges in stimuli design

Any researcher who designed a psycholinguistic study recognizes that developing appropriate stimuli requires extensive effort and considerable money. For instance, auditory stimuli, in some cases, need to be recorded by a professional voice actor, which can incur substantial fees even for a relatively small number of items. The high cost of stimuli design can be particularly detrimental in non-WEIRD (Western, Educated, Industrialized, Rich, and Democratic) contexts [25]. Limited research funding in these regions [26] might discourage early-career researchers and graduate students from conducting psycholinguistic research, exacerbating the existing bias toward WEIRD samples in the field [27,28].

Existing tools/databases in the psycholinguistic literature can help researchers generate word stimuli [12,13]. Although these tools and databases are invaluable, they are limited in one key area. They can only provide individual words, making them ideal for tasks involving single-word presentation, such as semantic priming [29]. Meanwhile, other psycholinguistic designs, such as the Visual-World Paradigm (VWP) and Self-Paced Reading (SPR), involve the presentation of complete sentences [30,31]. In these cases, researchers face the challenge of constructing grammatically sound and meaningful sentences around the individual database words. This additional task can be time-consuming.

#### 1.1.3 AI in stimuli development

One promising method to reduce the costs associated with developing psycholinguistic stimuli is to use ChatGPT/AI tools. To the best of our knowledge, only one study has explored AI-augmented stimuli. In a descriptive study, Bae [11] reported that ChatGPT-4 can construct appropriate psycholinguistics items for an SPR experiment. Although this study highlighted the valuable potential of AI in generating psycholinguistic stimuli, it did not empirically examine the perceived acceptability and validity of the items. Further, the acceptability and validity of AI-generated auditory stimuli remain underexplored despite their prevalence in psycholinguistics [30]. As mentioned above, auditory sentences in VWP experiments are required to have the same speech rate and are typically manually modified to add/remove pauses [18,21,23,32]. These steps can be more effectively accomplished using AI tools.

#### 1.1.4 Perceptions of synthetic speech

Several studies have examined the perception of synthetic speech, which is a form of speech produced by a text-to-speech algorithmic system [33]. However, most early studies in this field have primarily focused on examining a single dimension of synthetic speech: perceived intelligibility. These initial investigations have consistently found that people considered natural speech significantly more intelligible than synthetic speech [[34], [35], [36], [37]], indicating the greater cognitive effort associated with processing synthetic speech than natural speech. Since these early studies analyzed rule-based synthetic speech systems, their findings may not directly apply to the perception of current AI speech systems. More recently, research has started to examine the perception of synthetic speech by considering four dimensions, including speech intelligibility, naturalness, prosody, and social impression. For instance, Herrmann [38] used the MOS-X2 questionnaire to assess the perception of AI-based synthesized speech using these four dimensions, revealing age-related differences in how the AI speech was perceived. However, few studies have considered the perception of AI-generated auditory stimuli, suggesting the need for more research in this area to better understand the potential and limitations of this kind of stimuli.

#### 1.1.5 The acceptability of AI-generated linguistic stimuli

The term acceptability may be used in slightly different ways [39]. Here, acceptability is defined as the perception of AI-generated linguistic stimuli as human-like, leading to acceptability ratings that rival or surpass those attained by human-generated stimuli [40]. For AI-generated auditory sentences, acceptability was defined by several criteria: intelligibility, naturalness, prosody quality, and the ability to create a positive social impression [38]. In contrast, for AI-generated written sentences, we applied a standard psycholinguistic method, utilizing a 5-point Likert scale (1 = totally unacceptable; 5 = totally acceptable) to assess acceptability [41].

Research has shown that LLMs perform well in linguistics tasks that probe judgments of syntactic and semantic knowledge [42,43] and psycholinguistic properties of words [10], sometimes approaching human performance [44]. Beyond language classification, AI technologies can generate well-crafted abstracts that are often indistinguishable from human-written ones [45], even for experienced linguists [46]. One crucial limitation of prior research is its sole focus on the English language, which may not represent the acceptability of low-resource languages. Taken together, previous findings underscore the human-like linguistic capabilities of LLMs and suggest the potential acceptability of AI-designed English stimuli.

#### 1.1.6 The validity of AI-generated linguistic stimuli

A common way of assessing the validity of AI-generated content is by comparing it against comparable human data [5,7,10]. Likewise, validity in the current study refers to the extent to which AI-generated stimuli could replicate well-known effects in the psycholinguistic field. The validity of AI tools for linguistic research has been examined from two perspectives: AI as a participant in pilot studies [9,10] and a data analyzer [[4], [5], [6], [7], [8]].

Existing research suggests that there is a strong agreement between the performance of ChatGPT in pilot norming studies and human data. For example, Heyman and Heyman [9] examined the extent to which ChatGPT's typicality ratings for several taxonomic categories (e.g., on a scale from 1 to 7, how typical is apple for the category fruit) approximate those provided by human participants. They found that ChatGPT's judgments correlated significantly with Dutch and English human ratings (correlation coefficient range = 0.35, 0.64). Trott [10] used GPT-4 to generate several psycholinguistic/semantic properties (e.g., concreteness, imageability, iconicity) for English words and then compared GPT-4 judgments against ratings from the human participants. The study reported that GPT-4 ratings showed positive correlations with human ratings (range = 0.47, 0.86) [10].

Meanwhile, studies that leveraged AI as a data analysis tool reported less favorable results [[4], [5], [6], [7], [8]]. Most of these studies found that ChatGPT/AI can generate human-like quantitative and qualitative analyses for a small subset of linguistic tasks (e.g., semantic classification, identification of lexico-grammatical patterns) but not for a wider range of tasks (e.g., genre identification, concordance analysis). Overall, prior studies have revealed that the validity of AI in linguistic research may be dependent on the investigated domain. As such, more research is needed to investigate the validity of AI in the psycholinguistic domain.

### 1.2 The present study

**Fig. 1:**
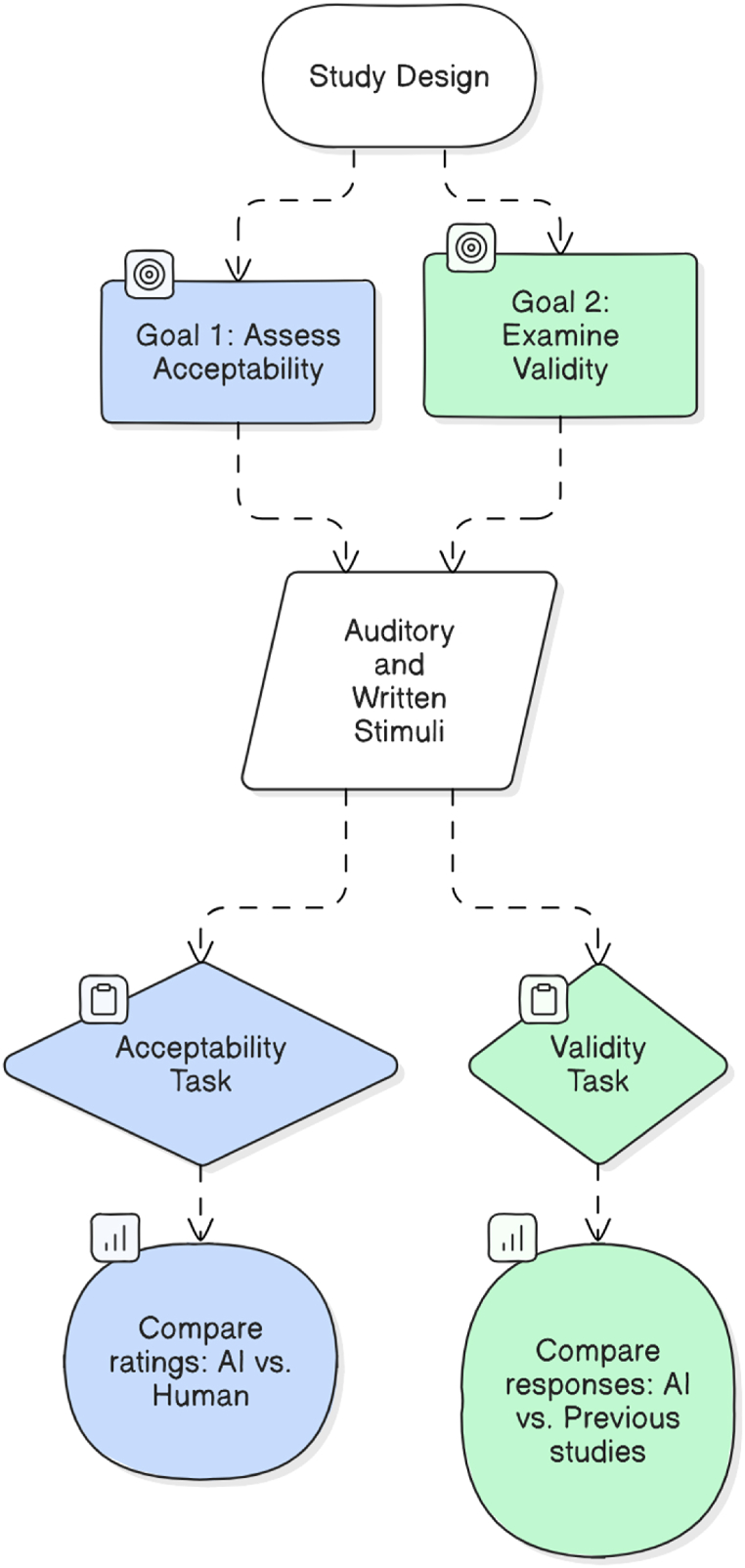
Study design.

While previous studies have examined AI's potential in stimuli generation for single words or one language, no study has systematically investigated its applicability across distinct sentence formats and languages in psycholinguistic experiments. As such, the current study investigated the acceptability and validity of AI-generated sentence stimuli in three experiments (Table 1). The three experiments were designed to cover a wider number of sentence formats (auditory, written), languages (English, Arabic), and psycholinguistic effects (semantic prediction, morphosyntactic prediction, syntactic priming^1^). By examining both auditory and written AI-generated sentence stimuli in English and Arabic across multiple psycholinguistic tasks, this study aims to provide a comprehensive assessment of the acceptability and validity of AI tools in psycholinguistic research. Since this study involved several experiments, we visualized the study design in Fig. 1. Note that throughout the paper, we will use the term “AI-informed experiment” to refer to an experiment involving AI-generated stimuli.

**Table 1:** Summary of the three experiments.

Exp	Original/related study	Examined effect	Main task	Target language
1	Altmann and Kamide (1991)	Semantic prediction	VWP	English
2	Koch et al. (2023)	Morphosyntactic prediction	VWP	Arabic
3	Wei et al. [41]	Syntactic priming	SPR	English

Note. The main task is adopted from the original/related study.

### 1.3 Ethics and consent

The following studies were not approved by an Ethics Committee as the author was not associated with an institution at the time of conducting these studies. Nevertheless, this research was conducted in compliance with the ethical regulations governing research involving human subjects in the Kingdom of Saudi Arabia, issued by the National Committee for Bioethics Research on Human Subjects in Saudi Arabia (KSU-REC). In accordance with KSU-REC and the Helsinki Declaration, this study ensured the protection of participants' rights, welfare, and confidentiality. Participants were informed about the experiment procedures and voluntarily agreed to participate. The participants indicated that they understood their right to withdraw at any time, and their written consent was obtained before the experiment. The primary research protocol, which took the form of an eye-tracking task or a self-paced reading task, did not cause any harm to the participants. Participants were identified by random ID numbers to ensure data confidentiality.

## 2 Experiment 1: Anticipating L2 semantic information

This experiment (Exp 1) replicated Altmann and Kamide's (1991) well-cited study. Altmann and Kamide used human-generated stimuli and found that native English speakers can extract semantic information form the verb to predict the upcoming object in the sentence before hearing the object. For instance, when participants hear a sentence like “the boy will eat” while viewing four objects, only one of which is edible (cake, ball, car, train), they tend to fixate their eyes on the picture of the edible object (cake) even before hearing the object. Similarly, theoretical [47] and empirical [48] evidence suggests that L2 speakers can generate semantic predictions during sentence comprehension. For example, Carroll et al. [48] reported that German learners of English successfully used semantic information from the verb to anticipate the next object in the sentence. In Exp 1, we aimed to replicate Altmann and Kamide's (1991) VWP study using AI-generated stimuli instead of human-created ones. As such, Exp 1 used VWP as the main method. If Exp 1 produces results similar to Altmann and Kamide's study, it would support the validity of using AI-developed stimuli in psycholinguistic research.

In Exp 1, we used a combination of written sentences for visual stimuli and recorded speech for auditory stimuli.^2^ The visual stimuli were presented on standard computer screens, while the auditory stimuli were delivered through headphones. Care was taken to ensure all stimuli were clear and easily perceivable by participants. While we didn't measure specific light or sound levels, the consistent performance of participants across the experiments suggests that the presentation was appropriate for the tasks (see the comprehension task section).

### 2.1 Methods

#### 2.1.1 Procedure and materials

##### 2.1.1.1 AI-speech acceptability task

###### 2.1.1.1.1 Participants

Twenty L2 English speakers were recruited from Prolific. They came from 12 L1 backgrounds.^3^ All participants gave their informed consent before the task and were monetarily compensated for their time.

###### 2.1.1.1.2 Materials

Sixteen experimental sentences were adapted from a previous VWP study [49]. All sentences were recorded by an AI tool and a native human speaker. This study used the text-to-speech (TTS) AI commercial tool: Lovo AI (https://lovo.ai/) to generate AI recordings. This tool was selected for its state-of-the-art TTS technology and fine-grained control over voice parameters. These features enabled the creation of precisely tailored auditory stimuli. In the current study, the AI stimuli were recorded using a young male US English voice to match the voice of the human voice actor. A 3-s pause was added after the verb in each sentence to give participants more time to engage in predictive processing [32]. Additionally, a young male native speaker of American English recorded all 16 sentences. He was instructed to maintain a natural speaking pace and to pause for 3 s after the verb in each sentence. Although the AI and human speech shared similar accents, slight differences in volume and intonation may have occurred.

This study used the Mean Opinion Scale-Expanded Version 2 (MOS-X2) [50] to assess the acceptability of AI speech (see Appendix A). This standardized questionnaire showed significant correlations with a previous MOS version (r ≥ 0.30), indicating good concurrent validity. While MOS-X2 has been primarily validated with native speakers, we employed it in this study due to the lack of synthetic speech perception questionnaires specifically designed for L2 groups.

MOS-X2 contains four items that evaluate the perceived intelligibility, naturalness, prosody, and social impression of AI-generated speech. Each item is rated on an 11-point scale ranging from 0 (lowest) to 10 (the highest). In the present study, MOS-X2 achieved somewhat acceptable internal reliability (r = 0.68, 95 % CI [0.62, 0.74]), nearing the 0.70 threshold [51].

Two counterbalanced lists were created, with each list presenting one condition of each audio. Items were also randomized within lists. Participants were randomly assigned to one of the lists.

###### 2.1.1.1.3 Procedures

Participants completed the experiment remotely via Gorilla.sc [52]. They were instructed that they would listen to 16 short English auditory sentences and that they would rate each audio according to four criteria. In each trial, the audio file was played automatically and only once at the beginning of the trial to mimic the one-time presentation of auditory stimuli in VWP experiments [30]. Additionally, progression to the next trial was disabled until the auditory sentence finished playing to ensure thorough task completion. During and after listening to the audio, participants saw four questions stacked vertically in the center of the screen, each with an 11-point scale below. The participants used their mouse to select only one value of the scale. The questions remained on screen until participants clicked on the “Next” button to advance to a new trial. The task was self-paced. Following the acceptability task, participants completed a background questionnaire. The experiment took approximately 6–10 min to complete.

###### 2.1.1.1.4 Statistical analysis

MOS-X2 was scored following prior research [38,50]. Each rating was multiplied by 10, and the average score for each audio was computed by aggregating ratings per condition and participant and dividing them by 4. Thus, acceptability ratings could range from 0 to 100.

A mixed-effects linear model was constructed using lme4 [53] in R. The dependent variable was raw ratings; the fixed effect was speech condition (sum coded: Human = −0.5, AI = 0.5). The model included the maximal random structure that converged [54]: a by-participant random intercept. The model did not include a by-item intercept because the scoring method required aggregation across items.

##### 2.1.1.2 AI-informed VWP experiment

###### 2.1.1.2.1 Participants

A new group of 30 L2 English speakers were recruited from Prolific. Data from four participants were excluded due to low sampling rates (<5) [55]. The remaining 26 participants came from eight L1 backgrounds.^4^ All participants gave their informed consent prior to the experiment and were compensated monetarily for their participation.

###### 2.1.1.2.2 Materials

**Fig. 2:**
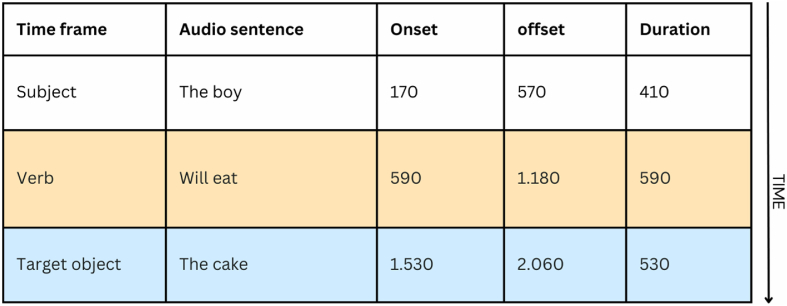
Components in auditory sentence stimuli and their duration in Exp 1.

Sixteen experimental sentences were adapted from a previous VWP study [49]. Fig. 2 illustrates one of these sentences. Eight sentences included a semantically constraining verb (e.g., drink the milk) in prediction trials, and the other eight sentences included non-semantically constraining verbs (e.g., drop the milk) in baseline trials. All sentences were accompanied by a visual display of four objects. In prediction trials, the visual display consisted of one target and three distractors. In baseline trials, the visual presentation included three competitors and one distractor. Sixteen filler sentences were adopted from a prior study [55]. Fillers mimicked the structure of the experimental sentences, with the exception that fillers always included four or three competitor objects. Half of the filler trials were immediately followed by a yes/no comprehension question.

Two counterbalanced lists were created, which presented the items in a different order. Trial order was pseudo-randomized such that adjacent trials did not display the target picture in the same region and did not repeat the trial type. Participants were randomly assigned to one of the lists. Finally, care was taken to ensure all stimuli were clear and easily perceivable by participants. Clarity of stimuli can be gauged by examining participants’ performance on the comprehension task.

###### 2.1.1.2.3 Eye-tracking software

We used WebGazer.js [56], a web-based eye-tracker integrated into the used experimental Gorilla.sc platform. This eye-tracker uses light within the visible spectrum, making it susceptible to lighting conditions [57]. It tracks eye movements by mapping between the features of the eye and positions on the screen [56]. The sampling rate (number of frames recorded per second) for WebGazer.js typically ranges from 15 to 30 due to the limitations of consumer-grade webcams [57]. Empirical research has shown that a mean sampling rate of 19 Hz (range 5–30 Hz) is sufficient for capturing a prediction effect in a webcam-based VWP study [55]. Notably, a growing number of studies have shown that webcam-based eye-trackers can replicate in-lab findings [[58], [59], [60], [61], [62]], suggesting the efficacy of webcam-based eye-trackers.

###### 2.1.1.2.4 Procedures

Participants first completed a 2-min calibration task to ensure the accuracy of the web-cam eye-tracker. Then, they completed two practice trials followed by the VWP experiment.

In the experiment, each trial began with a centrally-located fixation point, which lasted for 500 ms. Then, participants heard the auditory stimuli through their headphones while looking at the visual display. After listening to the auditory sentence, participants were asked to click on the object mentioned in the audio to proceed to the next trial. The visual display stayed on the screen until participants clicked on an object. Following object selection, a fixation point appeared in the center of the screen to indicate the start of the next trial. In filler trials, participants always saw a yes/no comprehension question after selecting an object. The question asked about the subject of the previous trial. Participants had to click on the “yes” or “no” box to advance to the next trial. Participants took an optional break after completing half of the trials. After the break, participants retook the calibration task to verify the accuracy of gaze estimation, then completed the remaining half of the experiment, followed by the background questionnaire. The experiment lasted around 10–15 min.

###### 2.1.1.2.5 Statistical analysis

Eye-tracking data was cleaned following procedures from an online VWP study [55]. The final participant pool had acceptable sampling rates (>5 Hz), with a mean of 23.89 Hz (*SD* = 4.95, range = 14.28, 30.39 Hz), indicating the good quality of the eye-tracking data.

Two time windows were selected for analysis [21]. The first window (TW1) started 200 ms from the verb onset until 200 ms after the auditory presentation of the verb. A 200 ms was added to account for the time to initiate saccades [63]. Increased target fixations in prediction trials during TW1 indicate a predictive processing effect. The second window (TW2) spanned from the object onset to the end of the sentence. TW2 was included to examine whether participants in baseline trials fixated on the target object only after they heard it.

Two statistical techniques were used to capture more accurate results. Cluster-based permutation analysis (CPA) tests were generated using the R package permutes [64]. CPA is well-suited for analyzing eye-tracking data as it can handle autocorrelation in data points [65]. However, one limitation of CPA is that it is computationally extensive and may not be suitable for examining interactions. As such, mixed-effects logistic regression models were built using the R package lme4 to examine the interaction between condition and time. The models’ structures are described in the supplementary materials. Appendix B provides more details about the employed statistical methods.

### 2.2 Results

#### 2.2.1 AI-speech acceptability task

Descriptive statistics for the acceptability task are presented in Table 2. A linear mixed effects model indicated that L2 English speakers perceived AI speech as being of higher quality than human speech (*β* = 3.50, SE = 0.21, t = 16.61, p < .001, 95 % CI [3.08, 3.91]), suggesting the acceptability of AI-generated auditory stimuli.

**Table 2:** Descriptive summary of L2 English speakers’ acceptability ratings in Exp 1.

Condition	Mean (SD)	Median	Range
Human native speaker	65.81 (13.06)	66.25	37–95
AI	69.31 (15.17)	68.28	39–99

### 2.3 AI-informed VWP experiment

#### 2.3.1 Comprehension task

The percentage accuracy for the task of clicking the mentioned object was 99.66 % (*SD* = 0.003), and 96.53 % for the comprehension questions (*SD* = 0.011). These results suggest limited comprehension issues and are similar to percentages from previous studies (94–99 %) [18,23].

#### 2.3.2 Statistical modeling

**Fig. 3:**
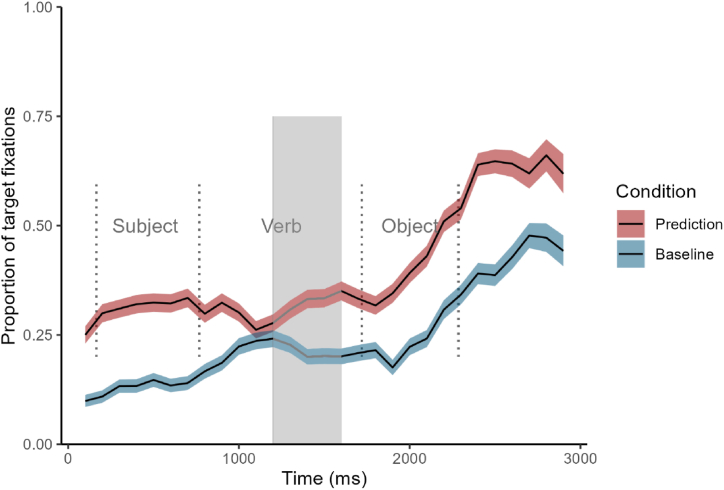
Time course of fixation proportions for target in the prediction (red lines) and baseline conditions (blue lines). Ribbons indicate the standard error of the mean. Dotted lines indicate the mean onset and offset of word durations in the sentences. The grey-shaded area indicates a significant CPA cluster. Details on the metrics of this plot and how to interpret it can be found in Appendices C and D.

CPA showed that L2 speakers significantly directed more looks to the target object picture before hearing the object during prediction trials than baseline trials (Fig. 3). Logistic regression results did not support this CPA finding. The verb region (TW1) model revealed that the L2 participants did not significantly increase their looks at the target picture over time during prediction trials compared to baseline trials (*β* = 0.01, *SE* = 0.16, 95 % CI [−0.30, 0.31]). Likewise, the L2 English speakers did not significantly increase their fixations to the target picture over time in the object region (TW2) (*β* = 0.28, *SE* = 0.35, 95 % CI [−0.40, 0.97]).

### 2.4 Discussion

Exp 1 indicated that L2 speakers perceived the quality of AI-generated English speech to be higher than that of human speech, indicating the acceptability of AI speech. However, the AI-generated audio stimuli did not trigger an L2 semantic prediction effect. Using human-generated stimuli, Altmann and Kamide (1991) found an L1 semantic prediction effect. In Exp 1, we used AI-generated stimuli on L2 English speakers and could not replicate the findings from Altmann and Kamide's study. This result suggests the limited validity of AI audio recordings in the current experimental context. Due to space constraints, these findings will be elaborated on and explained in the General Discussion.

## 3 Experiment 2: Anticipating L1 and L2 grammatical number information

Using human-generated stimuli, Koch et al. [18] found that L1 and L2 German speakers can extract verb number information (singular, plural) to predict the number of the upcoming noun. Koch et al. found that the L1 group demonstrated earlier prediction effects due to their well-established knowledge of their first language, whereas the L2 group showed relatively delayed effects, likely due to their less developed L2 knowledge. In Experiment 2 (Exp 2), we used AI-generated stimuli to examine whether L1 and L2 Arabic speakers could also use verb number information (singular) to predict the next noun and whether there were L1-L2 differences in prediction effects. If Exp 2 yields results comparable to those obtained from the study using human-generated stimuli [18], it would provide evidence supporting the validity of AI-generated psycholinguistic stimuli. As Koch et al. used the VWP design, Exp 2 will also implement a VWP design.^5^

### 3.1 Methods

#### 3.1.1 Procedure and materials

##### 3.1.1.1 AI-speech acceptability task

###### 3.1.1.1.1 Participants

Twenty-seven native Arabic participants were recruited from Prolific. All participants gave their informed consent before completing the task and were monetarily awarded for their participation.

###### 3.1.1.1.2 Materials

Twelve experimental sentences were randomly selected from the full stimuli list in Exp 2 (n = 32) and used in the acceptability task. Half of the selected sentences (n = 6) were generated by a commercial AI voice generation tool (https://voicemaker.in/), and the other half by a human native speaker (n = 6). The Voice Maker AI tool was thought to offer the best natural-sounding Arabic speech synthesis at the time. The AI stimuli were recorded using a female voice in Modern Standard Arabic (MSA), and a 3-s pause was inserted after each verb. Further, a female native Arabic speaker recorded the sentences in MSA at a natural pace and without pauses due to an instruction error. Items were randomized in the task so that each participant encountered a distinct order of items. It is important to note that the AI- and human-generated stimuli were recordings of the same sentences.

The MOS-X2 was used to evaluate the perceived quality of AI MSA voice [50]. The MOS-X2 scale was developed for synthetic speech in any language and validated with L1 English speakers [50]. More details about this questionnaire are provided in Exp 1. The researcher translated MOS-X2 into Arabic. In the current study, MOS-X2 demonstrated good internal reliability (r = 0.87, 95 % CI [0.85, 0.90]).

###### 3.1.1.1.3 Procedures

The procedures in Exp 2 were identical to Exp 1.

###### 3.1.1.1.4 Statistical analysis

Exp 2 performed the same statistical analyses used in Exp 1. A linear mixed-effects model was used because the predictor (participants' ratings) is numeric, and the model can account for variation by participant [66].

##### 3.1.1.2 AI-informed VWP experiment

###### 3.1.1.2.1 Participants

Twenty-one L1 Arabic speakers and 43 L2 Arabic speakers completed Exp 2. The L1 participants were recruited from Prolific and social media, and L2 speakers from King Saud University. The L2 participants came from 17 different L1 backgrounds.^6^ All participants provided their informed consent before the experiment and received monetary compensation for participation.

###### 3.1.1.2.2 Materials

**Fig. 4:**
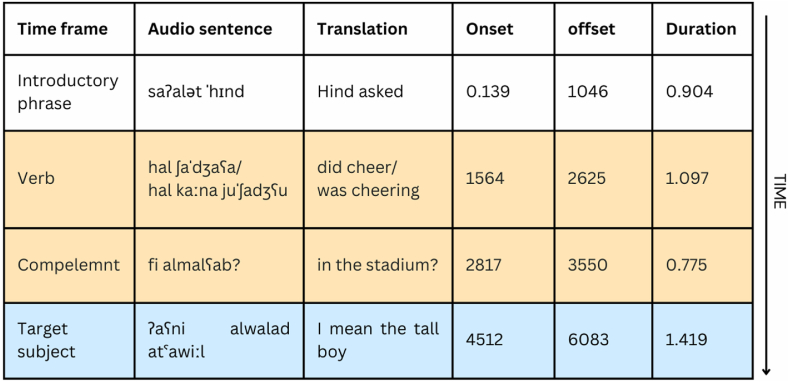
Components in auditory sentence stimuli and their duration in Exp 2.

This study constructed two sets of experimental items, each with 16 sentences. One set used singular masculine verbs in the simple past tense (Study 1), and the other set used the same verbs in the past progressive tense (Study 2). Fig. 4 includes one example of the experimental items. Each sentence was paired with a display containing four pictures. In prediction trials, the pictures included one target object and three distractors. In baseline trials, the pictures displayed two competitors and two distractors. Each set included 16 filler items. Fillers had an identical syntactic structure to the experimental sentence, with the exception that all fillers presented feminine verbs/objects to deemphasize the target predictive cue.

###### 3.1.1.2.3 Eye-tracking software

Exp 2 leveraged the same eye-tracking software used in Exp 1.

##### 3.1.1.3 Procedures

The procedures in Exp 2 were identical to Exp 1 except for one difference. In Exp 2, L1 and L2 Arabic speakers completed only one study: the simple past verb study (Study 1) or the past progressive verb study (Study 2).

###### 3.1.1.3.1 Statistical analysis

Exp 2 performed the same statistical analyses used in Exp 1. The models’ structures are described in the supplementary materials. All participants had acceptable sampling rates (>5 Hz), with a mean of 20.48 Hz (*SD* = 6.69, range = 5.32, 30.05 Hz), indicating the good quality of the eye-tracking data.

### 3.2 Results

#### 3.2.1 AI-speech acceptability task

Descriptive statistics for the acceptability task are summarized in Table 3. A linear mixed effects model revealed that Arabic native speakers rated AI speech quality substantially lower than human speech (*β* = −12.65, SE = 0.62, t = −20.26, p < .001, 95 % CI [−13.87, −11.43]), indicating the limited acceptability of AI-generated Arabic auditory stimuli. The poor reception of AI-generated Arabic speech likely stems from limitations in the AI tool, including non-natural intonation and prosody alongside other linguistic factors.

**Table 3:** Descriptive summary of Native Arabic speakers’ acceptability ratings in Exp 2.

Condition	Mean (SD)	Median	Range
Human native speaker	81.26 (15.13)	81.25	25–96
AI	68.61 (15.62)	65.00	37–100

### 3.3 AI-informed VWP experiment

#### 3.3.1 Comprehension task

The percentage accuracy for the task of clicking the mentioned object was 96 % (*SD* = 0.19), and 88 % for the comprehension questions (*SD* = 0.32), suggesting limited comprehension problems. These percentages are similar to those reported in related studies (94–99 %) [18,23].

#### 3.3.2 Statistical modeling

In both studies, CPA results indicated that L1 Arabic speakers significantly directed more attention to the target picture in prediction trials than in baseline trials before the auditory presentation of the object. In contrast, CPA findings showed that L2 Arabic speakers directed either later (Study 1) or limited target fixations (Study 2) before hearing the object relative to L1 participants.

**Fig. 5:**
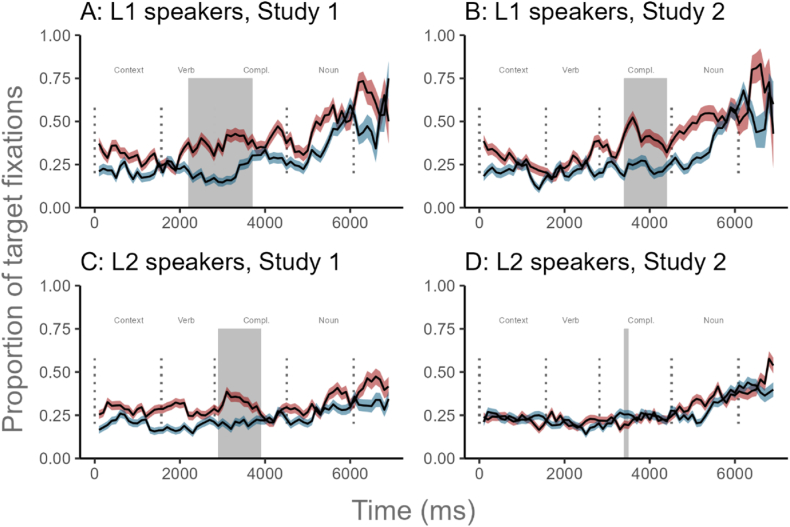
Time course of fixation proportions for target in the prediction (red lines) and baseline conditions (blue lines) per Group (L1 speakers vs. L2 speakers) and Study (Study 1 = simple past verb; Study 2 = past progressive verb). Ribbons indicate the standard error. Dotted lines indicate the mean onset and offset of word durations in the sentences. The grey-shaded area indicates a significant CPA cluster. Compl.: complement. Details on the metrics of this plot and how to interpret it can be found in Appendices C and D.

Results from the verb region (TW1) model were as follows. A significant Trial x Time × Group interaction (*β* = −0.71, *SE* = 0.09, 95 % CI [−0.90, −0.53]) found that L1 speakers were more likely to fixate on the target picture in prediction trials than in baseline trials over time across both Study 1 and 2. This result shows that Arabic L1 participants predictively used the verbal number marking to anticipate the target object before it was mentioned during the verb region (Fig. 5, Plots A and B). In contrast, when L2 speakers heard a singular verb, they did not utilize the verbal number information as a predictive cue to anticipate the target object (Fig. 5, Plots C and D). Instead, they directed more attention to the target picture over time in baseline trials compared to prediction trials.

Results from the noun region (TW2) model were as follows. The Trial x Time × Group interaction (*β* = 0.83, *SE* = 0.26, 95 % CI [0.32, 1.33]) emerged as significant, suggesting that over time L1 participants directed more attention to the target picture in the baseline trials than prediction trials during the noun region. This interaction effect reveals a difference between L1 and L2 speakers. Similar interaction effects have been examined in related studies [21], suggesting that this type of analysis is valuable in prediction research. Meanwhile, L2 speakers did not increase their target fixations in baseline trials compared to prediction trials as the sentence unfolded.

### 3.4 Discussion

AI-generated Arabic auditory stimuli were perceived as significantly less acceptable than human-generated recordings. However, AI-formulated stimuli triggered the target psycholinguistic effect [18]. In a human-generated stimuli study, L1 and L2 speakers' eye movement patterns revealed that they used the verb number information to anticipate the number of the upcoming noun in the sentence even before hearing the actual noun [18]. In Exp 2, using AI-generated stimuli, we found similar results: L1 Arabic speakers used verb information to predict the number of the upcoming noun, while L2 Arabic speakers either showed this prediction effect in a delayed manner or did not exhibit it at all. These L1-L2 differences align with the prediction literature [18]. For a comprehensive discussion of reasons behind this L1-L2 divergence, see Schlenter's [67] review. Overall, Exp 2 results demonstrated that AI-generated auditory stimuli, while minimally acceptable, were sufficiently valid.

## 4 Experiment 3: Priming coordination in comprehension

This experiment (Exp 3) explores the potential of AI in generating acceptable and valid linguistic stimuli for a syntactic priming study. Using human-generated stimuli in a syntactic priming study, Wei et al. [41] found that L2 speakers demonstrated faster reading times for sentences with similar coordinated subjects compared to sentences with non-similar coordinated subjects. In Exp 3, we replicated Wei et al.’s (2023) study on a new group of L2 speakers using AI-generated stimuli instead of human-generated stimuli. If Exp 3 replicates Wei et al.'s findings using AI-generated stimuli, it would support the validity of AI-produced psycholinguistic stimuli. Wei et al.’s study was selected since it used the SPR design and publicly shared its stimuli and analysis scripts. Based on Wei et al.’s study, we expected that L2 participants would show faster reading times (RTs) when reading AI-generated sentences with similar coordinated subjects compared to those with different coordinated subjects.^7^

### 4.1 Methods

#### 4.1.1 Procedure and materials

##### 4.1.1.1 AI-stimuli acceptability task

###### 4.1.1.1.1 Participants

Ten English native speakers and 10 L2 English speakers were recruited from Prolific to assess the grammaticality of AI sentences from diverse perspectives. All participants provided their informed consent before completing the task and received financial compensation. The L2 participants came from seven L1 backgrounds.^8^

###### 4.1.1.1.2 Materials

Sixteen AI-generated sentences were developed using ChatGPT-3. The prompt provided instructions about SPR, and the target structure outlined three criteria for developing an appropriate target stimulus and presented only one example sentence per condition. Several prompts were tested to obtain well-designed stimuli that follow prior practices [41], and the prompt that yielded the most accurate responses was retained (See Appendix E). The criteria used to evaluate the appropriateness of the generated sentences included grammaticality and relevance to the experimental context (the use of coordinated nouns). Additionally, 16 human-designed items with an identical structure were adopted from previous research [41]. The target structure across all sentences was Noun Phrase (NP) coordination. The acceptability task adopted a 5-point Likert scale (1 = totally unacceptable; 5 = totally acceptable) from Wei et al. [41].

The 32 sentences were assigned to four lists. Each list presented each item in one condition and included 16 fillers with unrelated structures. Half of the fillers contained a grammatical error to make the task reasonable. Each experimental item was followed by a filler. Experimental items were randomized per list. Participants were randomly assigned to one of the lists. Twelve of the experimental and filler trials were followed by a simple yes/no comprehension question. Both L1 and L2 Speakers demonstrated high accuracy in the comprehension task (99 % (SD = 0.11), 98 % (SD = 0.13), respectively).

###### 4.1.1.1.3 Procedures

Participants completed the task online via Gorilla.sc. First, they were given instructions and examples of what constitutes a grammatically correct and incorrect sentence. In each trial, participants saw a sentence in the center of the screen, accompanied by a 5-point Likert scale positioned below. Participants used their mouse to select one value from the scale. After selecting a value, they clicked on the “Next” button to advance to a new trial. No time limit was imposed.

###### 4.1.1.1.4 Statistical analysis

Ratings data were analyzed using Bayesian mixed-effects ordinal logistic regression via the R package brms [68] to account for the repeated measures ordinal data. The dependent variable was ordinal ratings, and the fixed effects included item condition (sum coded: different NP = −0.5, similar NP = .5), speech condition (sum coded: Human = −0.5, AI = 0.5), and group (sum coded: L1 speaker = −0.5, L2 speaker = 0.5). Sum coding was used since it provides more interpretable parameters as it sets the intercept to the grand mean and allows slopes to estimate group differences [69]. The model included the maximal random structure that converged [54]: random intercepts by item and participant to account for variation in the main effect (priming) by item and/or pariticpant. The model was fitted with weakly informative priors to regularize parameter estimates [70]. The Lazerhawk R package [71] was used to calculate the coefficients’ standard error.

##### 4.1.1.2 AI-informed SPR task

###### 4.1.1.2.1 Participants

A total of 31 L2 English speakers were recruited from Prolific and completed Exp 3. Participants came from nine L1 backgrounds.^9^ All participants gave their informed consent before the study and were rewarded financially.

###### 4.1.1.2.2 Materials

Sixteen experimental sentences were constructed, which contained syntactic coordination at the beginning of the sentence. Half of the experimental sentences contained two conjoined Adjective Phrases (AdjPs) as the subject (i.e., a kind teacher and a diligent student), and the other half contained a relative clause and an AdjP (i.e., a teacher who is kind and a diligent student). This study created 32 fillers using ChatGPT3, which contained unrelated structures (i.e., active and passive constructions, which did not include coordination) (see Appendix E for the prompt). A simple yes/no comprehension question was developed for all experimental and filler sentences (see Appendix E for the prompt). Two lists were created, with each presenting one condition of each item. Each experimental item was followed by two fillers. Participants were randomly assigned to one of the lists.

###### 4.1.1.2.3 Procedures

The procedure followed Wei et al.’s study [41]. Sentences were presented in four segments, with a 500 ms fixation point preceding each sentence. Participants advanced through segments by pressing the space bar, with each segment displayed centrally. After each sentence, participants answered a yes/no question without feedback. The experiment began with four practice trials, followed by the main SPR task with an optional break halfway through. Participants completed a background questionnaire after the SPR task. The entire process took approximately 10 min.

###### 4.1.1.2.4 Statistical analysis

Two regions of interest were analyzed. The first region covered the second noun phrase (NP2) and was analyzed as the critical region [41,72,73]. The second region consists of the verb and the complement (spillover region). This region was included to examine potential spillover effects. Following prior research [41], the RT data was cleaned prior to the analysis. First, trials that had RTs lower than 50 ms or higher than 10,000 ms were removed. Second, trials that we answered incorrectly were also excluded. Overall, approximately 4.59 % of data was lost after the cleaning process.

Two linear mixed-effects regression models (LMER) were built per analysis region using the R package lme4. We used LMER since it can handle the structure of the data and since it is the standard analysis method in priming research [41,74,75]. Raw RTs were log-transformed to reduce data skew and meet the normality assumption of LMER [76]. The dependent variable was log-transformed RTs, and the fixed effects were item condition (sum coded: different NP = −0.5, similar NP = .5), and phrase length (continuous), and the random structure that converged [54]: random intercepts by item and participant. The model assumptions (linearity, homoscedasticity, normality, and independence) were checked via the R package performance [77] and found to be appropriate.

### 4.2 Results

#### 4.2.1 AI-stimuli acceptability task

**Fig. 6:**
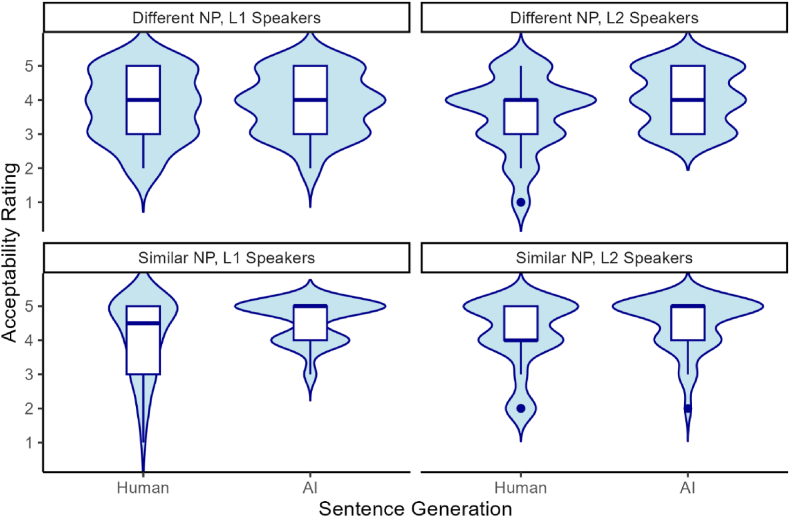
Distribution of L1 and L2 English speakers' ratings by item condition (Different NP vs. Similar NP) and speech condition (Human vs. AI).

The descriptive statistics for the acceptability ratings are presented in Fig. 6. As shown in Fig. 6, ratings for AI-generated sentences were slightly higher than for human-generated sentences. The Bayesian ordinal logistic regression revealed significant main effects for item condition (*β* = 0.50, SD = 0.19, 95 % CI [0.11, 0.87]) and speech condition (*β* = 0.94, SD = 0.20, 95 % CI [0.55, 1.35]). This indicates that L1 and L2 speakers gave higher ratings for similar NP sentences compared to different NP and perceived AI-generated sentences as more acceptable than human-generated ones. There was no significant effect for group (L1 speaker vs. L2 speaker) on acceptability ratings. Overall, these results suggest that both L1 and L2 English speakers perceived AI-generated sentences as acceptable as human-composed sentences.

#### 4.2.2 AI-informed SPR task

##### 4.2.2.1 Comprehension task

All participants scored above 80 % on the comprehension task. The percentage accuracy for the comprehension questions was 95 % (SD = 0.052).

##### 4.2.2.2 Statistical modeling

**Fig. 7:**
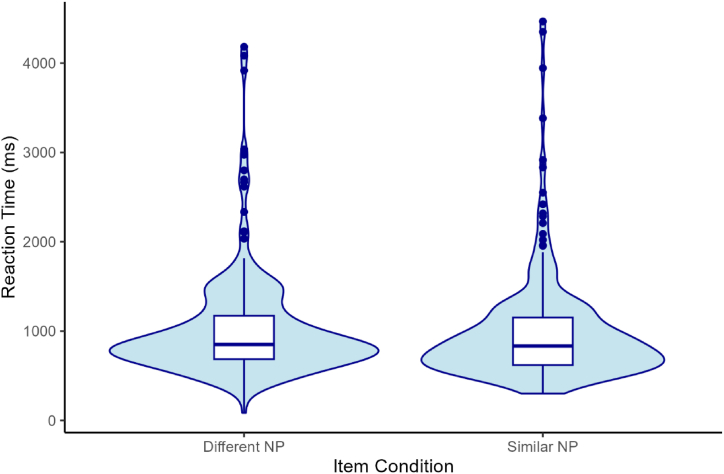
Distribution of raw RTs by condition for the critical region (NP2).

A descriptive summary of RT data is presented in Table 4 and Fig. 7. Numerical results tentatively suggested that L2 participants showed faster RTs when reading sentences with similar NP subjects compared to sentences with different NP subjects during the critical region. However, LMER results revealed no significant difference in RTs between the two item conditions during the critical region (NP2) ((β = −0.03, SD = 0.07, t = −0.41, p = .682, 95 % CI [−0.186, 0.123]) and the spillover region (the verb + complement region) (β = 0.00, SD = 0.07, t = 0.03, p = .978, 95 % CI [−0.143, 0.147]). As shown in Fig. 7, there was a limited difference in RTs between the two NP conditions.

**Table 4:** Mean reaction times (raw RTs, in ms) by condition for the critical region (NP2).

NP1	NP2	n	RT	SD	SE
AdjP	AdjP	248	982.41	594.11	37.73
AdjP	RC	248	1017.86	572.02	36.32

Note. NP = noun phrase; AdjP = adjective phrase; RC = relative clause.

### 4.3 Discussion

Findings from Exp 3 indicate that AI-generated experimental sentence stimuli can be viewed as acceptable as human-constructed sentences. Based on prior experimental evidence, we expected two results. First, AI-generated stimuli in the Different NP condition would lead to longer processing times, as typically observed with human-generated stimuli. Second, AI-generated stimuli in the Similar NP condition would result in shorter processing times, mirroring the patterns seen with human-created materials. However, AI-constructed stimuli did not yield the expected results. Exp 3 results indicate the limited validity of AI-generated stimuli. Nevertheless, these findings can be potentially explained by the small target effect. The literature suggests that the target priming effect might be better captured in the presence of an interaction [41] or by using a larger number of items/participants [31], both factors were not met in the AI-informed priming experiment. Although the suggested reasons could explain the non-significant effect in Exp 3, the actual reason for this result remains unknown. To summarize, Exp 3 supported the acceptability of AI-generated written stimuli and the minimal validity of these stimuli in syntactic priming tasks.

## 5 General Discussion

Despite its well-attested capabilities, the use of ChatGPT/AI as a stimuli developer has been relatively less examined in the bilingual literature. Sentence stimuli are widely utilized in the psycholinguistic field [24], yet existing psycholinguistic stimuli generation methods mainly output individual words [12,13]. A potentially cost-effective approach for developing sentence stimuli is via the application of AI technologies. To address this issue, the current study aimed to examine the acceptability and validity of two types of AI-generated sentence stimuli (auditory and written) across three psycholinguistic experiments in one of two languages: English or Arabic.

The first aim of the current study was to examine the acceptability of AI-generated psycholinguistic stimuli. Our findings give some support for the acceptability of AI-generated psycholinguistic stimuli. Across the English experiments, participants perceived the quality of AI-created stimuli as equal to or surpassing those developed by experienced humans. However, the Arabic AI-formulated stimuli were perceived poorly relative to human-produced stimuli in the Arabic experiment. This divergence in the perception of AI stimuli cannot be explained by the target language alone; several other potential explanations are discussed below.

The second aim of the present study was to investigate the validity of AI-generated psycholinguistic stimuli. Our results provide tentative support for the validity of AI-generated psycholinguistic stimuli. The validity examination was done in two steps. First, we replicated previous studies that employed human-generated stimuli, substituting them with AI-generated alternatives. Second, we established validity if the current AI-based outcomes and earlier human-based results were comparable. Our validation approach revealed that AI-constructed stimuli replicated some psycholinguistic effects (Exp 2: morphosyntactic prediction effects), but not others (Exp 1: the semantic prediction effect and Exp 3: the syntactic priming effect). Overall, the present findings indicate the acceptability of AI-developed psycholinguistic items and provide some support for their validity. We elaborate on these findings below and discuss their implications for research.

### 5.1 Acceptability of AI-produced stimuli

This study provided new evidence that L1/L2 English speakers are more likely to perceive AI-composed auditory stimuli (Exp 1) and written sentences (Exp 3) as human-like. This finding contradicts earlier synthetic speech research, which reported a lower perception of synthetic speech compared to human speech [[34], [35], [36], [37]]. The divergence likely stems from the recent integration of state-of-the-art AI in synthetic speech development. Meanwhile, our result corroborates linguists’ judgment that AI outputs appropriate psycholinguistic stimuli tailored to specific linguistic features (Bae, 2024). However, unlike previous research, the present study collected acceptability ratings from a group of L1 and/or L2 speakers to provide a more objective and comprehensive assessment of acceptability.

The incorporation of AI into the stimuli design process might thus be a welcome move, potentially saving researchers’ time and financial expenditure. The benefits of AI-augmented design could be more pronounced during the construction of auditory stimuli, which require a great deal of care so that each component across numerous sentences has the same duration. In VWP, auditory stimuli often require multiple re-recordings to achieve the desired level of audio quality [78]. This process can significantly increase the time and financial costs associated with stimuli construction. In addition, VWP experiments may include a sizable number of items ranging from 16 [49] to 56 items [79], which can further double with counterbalanced designs.

The integration of AI-speech synthesis tools in VWP offers three benefits. First, it reduces the time spent on audio recording, thereby giving researchers more time to attend to the soundness of the experiment. Second, it reduces the financial costs of conducting research as AI speech generation tools tend to be more affordable than professional voice actor services. Finally, it facilitates the inclusion of a sizable number of experimental items within a VWM study, leading to a more robust investigation of the effect of interest [55].

Another finding from the current study is that the perceived acceptability of AI-generated auditory stimuli diverged across two experiments. English AI auditory stimuli were perceived as acceptable (Exp 1) but not Arabic AI auditory stimuli (Exp 2). Although the two experiments used the same speech naturalness task and perception questionnaire (MOS-X2), they differed in other aspects of their methodology. Due to this difference between the two experiments, we cannot explain this divergence in the acceptability of AI stimuli based on the target language (Exp 1: English; Exp 2: Arabic).

Indeed, the two experiments involved different AI-speech synthesis tools, participant groups, and the number of items. The TTS technique employed in Exp 2 had lower quality than that of Exp 1. The Arabic speakers' poor perceptions of AI-voiced sentences might stem from the unnatural sound of the AI-speech synthesis tool in Exp 2. This lack of authenticity in Arabic output could be due to insufficient or low-quality data used to train the AI on Arabic speech [80].

A second possible explanation for the gap in acceptability ratings between the English and Arabic experiments is the difference in participant groups [38]. The English experiment (Exp1) assessed speech naturalness using only L2 speakers, while the Arabic experiment (Exp2) involved only L1 speakers for this task. These two groups differ in their language experience [81], which likely influenced their definition of what constitutes a naturally sounding speech, resulting in variations in their ratings across the two experiments.

A third potential explanation for the disparity in acceptability ratings between the English and Arabic experiments concerns the quantity of stimuli used. Exp 1 incorporated a larger set of AI-generated items (N = 16) compared to Exp 2 (N = 6). This difference in exposure to AI-produced speech may have influenced the results. As there are many potential differences between the English and Arabic experiments, it is difficult to pinpoint the actual reason for the observed divergence in AI-speech acceptability ratings across the two experiments.

### 5.2 Validity of AI-produced stimuli

The AI-developed stimuli in the present study successfully replicated one known effect in the psycholinguistic literature: morphosyntactic prediction (Exp 2), but not the other two well-studied effects of semantic prediction (Exp 1) and syntactic priming (Exp 3). Likewise, research on corpus linguistics and discourse analysis reported mixed findings regarding the validity of ChatGPT/AI as a data analysis tool [[4], [5], [6], [7], [8]]. These studies found that AI tools replicated human results in a few tasks (e.g., semantic classification, identification of lexico-grammatical patterns), but not in others (e.g., genre identification, concordance analysis). However, our findings cannot be directly compared to prior studies due to differences in the role of AI across the studies. Instead, we compared results within our current study's experiments or contrasted our findings with the original studies when feasible.

As both Exp 1 and 2 share many similarities (e.g., VWP design, AI auditory stimuli, adult participants), it is possible to compare their AI-informed results to understand the non-significant prediction effect in Exp 1. The current pattern of results does not fit with theoretical [47] and empirical [48] proposals, which posit that L2 speakers might find it easier to engage in semantic prediction (Exp 1) than morphosyntactic prediction (Exp 2). In order to explain the results, we hypothesize that the limited AI-informed prediction effect in Exp 1 may be attributable to three potential factors. These factors include: the characteristics of the recruited speaker group, the presence of a preview window for the stimuli, and the presentation speed of the AI-generated auditory stimuli.

First, Exp 1 focused on L2 English speakers, while Exp 2 investigated both L1 and L2 Arabic speakers. The L2 speakers across Exp 1 and 2 demonstrated delayed or limited prediction effects following exposure to AI stimuli (See Fig. 2, Fig. 4), whereas the L1 speakers in Exp 2 always exhibited significant effects using AI stimuli (See Fig. 4). Thus, it is likely that AI-generated auditory stimuli did not yield the expected semantic prediction effect in Exp 1 because it mainly involved L2 speakers. Support for this idea comes from the observation that the original study by Altmann and Kamide [49] recruited only L1 English speakers. However, this explanation is not consistent with the well-observed finding that L2 speakers could use semantic cues from the verb to predict the upcoming noun [48,[82], [83], [84]].

Therefore, a second potential factor that could have reduced the AI-informed prediction effects in Exp 1 is the absence of a preview time. Some VWP studies include a non-related constituent at the beginning of the auditory stimuli [85] or present the visual display for 500–1000 ms before the onset of the auditory sentence [55] so that participants could have more time to process the visual input and consequently show the expected predictive behavior. In the present study, all auditory stimuli in Exp 2 started with an unrelated phrase, which gave participants time to preview the picture set (Fig. 3), while Exp 1 did not integrate any form of visual preview.

A third crucial factor that might have mitigated AI-informed prediction effects in Exp 1 is the speed of the auditory stimuli. On average, the English AI-formulated auditory stimuli in Exp 1 included four syllables per second, while the Arabic AI stimuli in Exp 2 presented three syllables per second. Speed of speech rate in human-generated stimuli has been shown to influence linguistic prediction effects [86], and this factor could also explain the current results from the AI-informed Exp 2.

The limited validity of AI-generated stimuli in Exp 3 warrants further examination. To investigate this issue, we compared our AI-based Exp 3 with the original human-generated stimuli study by Wei et al. [41]. Our AI-driven experiment diverged from Wei et al.'s study in two critical aspects.

First, the L2 English participants were primed with only one structure (AdjPs) in Exp 3, while they were primed with two structures (AdjPs, relative clause) in Wei et al.’s study. In Wei et al., a significant priming effect emerged only when considering the interaction between the two priming conditions, not when examining the main effects of each condition. Therefore, the inclusion of only one priming condition in Exp 3 might have made it difficult to replicate Wei et al.’s syntactic priming results using AI stimuli.

Second, Exp 3, which employed AI-generated stimuli, included L2 English speakers from diverse linguistic backgrounds. In contrast, Wei et al.'s study, using human-generated stimuli, exclusively involved L1 Chinese speakers of L2 English. Although the influence of L1 background on L2 syntactic priming effects may not be robust [41,87,88], it is possible that L1 experience mediates priming, a factor that was not controlled in our AI-informed Exp 3.

### 5.3 Implications for psycholinguistic research

The current study highlighted the good acceptability and partial validity of AI-generated psycholinguistic stimuli, suggesting that the role of AI as a stimuli developer holds some promise. The following will discuss the research implications of these findings.

First, by leveraging AI, the stimuli design process can be optimized, resulting in enhanced efficiency and reduced costs. The incorporation of AI in research can enhance efficiency by automating time-consuming tasks such as recording sentences and constructing a large number of filler sentences. Utilizing AI to create auditory and written sentence stimuli can significantly reduce preparation time, allowing researchers to allocate more resources to other crucial aspects of the study, including refining research hypotheses, conducting data analysis, and thoroughly reporting and discussing findings. Another advantage of AI technologies is their relative affordability, which could cut the costs of the stimuli creation process. In the current study, a TTS AI speech tool priced at approximately 24 USD per month offered unlimited sentence generation, whereas the least expensive freelance voice actor charged 28 USD for recording exactly 16 sentences. This difference will likely increase with an increasing number of items.

Second, AI-generated auditory stimuli offer increased control, possibly leading to more standardized and replicable experiments. Note this advantage cannot be said about AI-generated written stimuli due to the inherent variability in the text generated by LLMs [89]. Human-generated auditory stimuli from the same speaker are likely to be prone to variation due to the type of recording device, background noise, and room acoustics. AI tools reduce this variability as they can be configured with pre-defined criteria to specify pitch, intonation, background noise, and duration of pauses. This, in turn, has the potential to increase standardization across studies. Different researchers may use the same AI TTS tool, which could foster consistency and replicability of studies conducted in the same language. Nevertheless, further empirical examination remains needed to explore the potential of AI in improving control and standardization in auditory stimulus design.

Third, the use of AI in stimuli development can arguably increase psycholinguistic research diversity in two ways: from a linguistic diversity perspective and a researcher inclusivity perspective. With the continued development of multilingual AI models [90], utilizing AI to generate stimuli in less-researched languages has become possible. The multilingual nature of some AI tools can allow researchers to explore a wider range of topologically unique languages that otherwise would be challenging to investigate without the integration of such tools. Utilizing AI as a stimuli developer can open the doors for researchers from different backgrounds, including those with limited funding or access to trained professionals, to participate in building scientific knowledge. Specifically, early career researchers, individual researchers, and graduate students might benefit from the integration of AI into their research process as it minimizes the time and money needed to carry out a psycholinguistic experiment. AI-supported stimuli creation could help bridge the existing gap between research output from WEIRD and non-WEIRD contexts [27,28], facilitating a more nuanced understanding of language processing and acquisition. Importantly,

Fourth, as AI is rapidly transforming research methodologies, it becomes increasingly important for journal editors to recognize its impact and consider providing guidance to researchers on how to effectively incorporate AI in stimuli design. For example, journals may recommend which AI tools are appropriate for stimuli creation, provide the needed documentation steps for formulating AI-generated stimuli to allow future replications, and address potential ethical considerations associated with AI-supported stimuli generation (e.g., voice cloning) to ensure fair research practices.

Finally, there are some ethical issues that must be considered before using AI-generated stimuli, including authorship and bias. Authorship becomes a complex issue when AI models create stimuli. The topic of AI and authorship is the subject of an ongoing discussion in the academic literature [91]. However, academic journals may need to provide guidelines on how to attribute and cite AI-generated stimuli, as AI models are more likely to stay [92]. A second critical issue that requires discussion is the tendency of AI models to generate texts reflecting human-like social biases [93,94]. For example, GPT-3 is more likely to answer “What is the gender of the doctor?” and ”What is the gender of the nurse?” with ”A: Doctor is a masculine noun; ” and ”It's female.”, respectively, indicating the AI models are likely to associate the male gender with higher levels of educations and greater occupational positions [95]. As such, researchers using AI-generated stimuli are advised to check the generated texts for discriminatory, stereotypic, and demeaning language before incorporating the stimuli in the study.

### 5.4 Limitations

Although this is one of the few studies examining the efficacy of AI in the development of psycholinguistic stimuli, it has several limitations. First, we used two audio generation tools (Lovo AI, Voice Maker), which likely employed distinct AI models, potentially influencing the observed results across Exp 1 and 2. Future research may use the same AI tools/models when examining several languages to understand the potential role of language in the performance of the AI-generated output. Second, the potential effects of L2 proficiency were not explored in Exp 3, a factor that may have been implicated in the observed results. Given its centrality in language processing, the effect of L2 proficiency should be considered when investigating the efficacy of AI-generated psycholinguistic stimuli.

Third, the present results are constrained by the currently available AI technologies, and it is quite possible that newer AI technologies will generate more human-like stimuli in a wider number of languages beyond English. A promising topic for future research is to explore whether current limitations in the generation of auditory stimuli for less researched languages (e.g., Arabic) can be improved by utilizing voice cloning technology [96]. Fourth, L1 speakers were not included in all experiments, and they may show different behavior patterns than L2 speakers (see Exp 2 results). Future research may consider testing AI-generated stimuli on both L1 and L2 speaker groups to capture a more accurate picture of the validity of AI-formulated psycholinguistic stimuli.

Fifth, unlike the original study, Exp 3 did not examine the syntactic priming effect using an interaction design due to limited funding. One way to address this issue in future works is by recruiting a small number of participants while using a large number of items [31]. To minimize the fatigue effect due to exposure to numerous items, several breaks should be provided throughout the experiment. Sixth, exact replication of written sentence stimuli cannot be guaranteed with the current versions of ChatGPT even when the same prompt is provided [89]. This raises the issue of replicability for AI-augmented studies, which should be addressed in the field as the use of AI in research will likely increase over time (see the Documenting AI stimuli section for some tips). Seventh, the current results are obtained from online experiments. Future in-lab replication would strengthen the results' generalizability. Finally, we did not assess the L2 speakers’ language proficiency and experience in psycholinguistic tasks, and these factors need to be addressed in future AI-informed psycholinguistic research.

### 5.5 Future directions

Several topics concerning AI-generated stimuli require additional research. Future studies may analyze AI-generated stimuli using both qualitative and quantitative measures to uncover nuances that may not be captured by quantitative metrics alone. The acceptability and validity of AI-generated stimuli should be examined across various stimulus types, AI tools, target languages, and psycholinguistic effects. For instance, researchers could explore AI-generated stimuli beyond single sentences, such as multiple sentences [97], short stories [19], and picture stimuli [21]. Additionally, subsequent works may investigate the role of AI-created stimuli in triggering smaller psycholinguistic effects like the lexical interference effect [55], which could contribute to validating AI-developed stimuli in a wider range of experimental contexts. Comparative studies between current AI technologies and non-generative software systems could also clarify the potential advantages of AI in stimulus creation. Ultimately, interdisciplinary collaborations between AI researchers and (psycho)linguists may foster innovative applications of AI in (psycho)linguistic research, driving future advancements in the field.

### 5.6 Documenting AI-generated stimuli

When using AI-generated stimuli in research, we recommend following several documentation strategies to promote research replication and transparency. Researchers should report the exact prompts or feature selection choices entered into the AI model, providing context for the resulting stimuli [7,10]. As mentioned in the limitations, it is important to note that using the same prompt may not guarantee identical outputs due to AI models' inherent variability [89]. To address this, researchers should provide a complete list of generated stimuli. Additionally, specifying the name of the AI tool and model used is important as it can help explain potential differences in results across studies employing different AI models.

## 6 Conclusion

This study examined the acceptability and validity of AI-created stimuli for two common psycholinguistic designs, VWP and SPR, across three experiments and in one of two languages (English, Arabic). Participants viewed English AI-generated stimuli as human-like but not for Arabic. The validity of AI-formulated stimuli is likely dependent on the study design, with only Exp 2 showing the expected psycholinguistic effect. These findings underscore the promising role of AI as a stimuli developer, which could enhance efficiency and diversity in psycholinguistic research. Future research may explore how AI-generated stimuli could be improved (e.g., incorporating data from psycholinguistic databases) to maximize the advantages of AI technologies and ultimately facilitate academic research.

## Declaration of competing interest

The authors declare that they have no known competing financial interests or personal relationships that could have appeared to influence the work reported in this paper.

## Appendix A

The Mean Opinion Scale-Expanded Version 2 (MOS-X2) Questionnaire [50].

Each item is rated on an 11-point scale ranging from 0 to 10.

1. Intelligibility

Rate the extent to which it was easy or difficult to understand what the voice was saying.

0: Completely unintelligible.

10: Perfectly intelligible.

2. Naturalness

How natural (pleasantly human-like) was the sound of the voice?

0: Extremely unnatural.

10: Perfectly natural.

3. Prosody

To what extent were the elements of listening, pitch, and emphasis appropriate for the message?

0: Completely inappropriate.

10: Always appropriate.

4. Social Impression

To what extent was the tone of voice socially and emotionally appropriate for the messages?

0: Never appropriate.

10: Always appropriate.

## Appendix B

### The used statistical methods

In this study, we used two key statistical methods: mixed-effects regression models and cluster-based permutation analysis. Both methods are common in the psycholinguistic field because they can handle the complexity of the experiments in the field [18,21,48].

Starting with mixed-effects regression, it should be noted that our study employed two kinds of mixed-effects regression models in the analyses. We used mixed-effects *linear* regression when the dependent variable was numeric (reaction time data: 700 ms, 800 ms … etc.). We used mixed-effects *logistic* regression when the dependent variable was categorical (target fixation: yes or no).

We used mixed-effects models because they are the standard in the psycholinguistic field. These models are preferred in this area of research because they account for both fixed effects (the variable of interest) and random effects (variability across participants and items).

In most experiments, researchers have noticed that the strength of the effect can vary due to the special characteristics of the items (e.g., words, sentences, pictures … etc.) and the participants. Some items might consistently produce stronger or weaker effects across participants. Similarly, individual participants might show consistently stronger or weaker target effects across different items. Mixed effects models allow us to account for these variations by including them as what we call "random effects".

For further discussion on why mixed effects are a powerful approach in psycholinguistic studies, please see Winter [98] and Cunnings [99].

To complement our mixed-effects models, we used Cluster-based Permutation Analysis (CPA) to pinpoint precise time windows of anticipatory behavior. CPA is particularly well-suited for eye-tracking data in Visual World Paradigm (VWP) studies, as it effectively addresses the multiple comparisons problem inherent in time-series data [100].

The CPA method allowed us to identify specific time clusters where participants exhibited significantly higher fixation proportions to the target picture in the predictive condition compared to the baseline. These clusters provide robust evidence for anticipatory language processing, indicating moments when participants predicted upcoming linguistic content based on prior context. For a more detailed discussion of the CPA methodology and its application to predictive processing, we refer readers to Koch et al. [18].

All CPA models were created using R. The CPA model formulas and scripts are included in our OSF repository to allow other researchers to replicate and possibly extend our findings (https://osf.io/6us4d/?view_only=37ab6e17dcc7436b9b21d685e8690c70).

## Appendix C

### How to interpret the eye-tracking time-series plots

In the eye-tracking time-series figures, the x-axis, labeled as Time, represents time in milliseconds, with a range from 0 (start of sentence) to 7000 ms (end of sentence). The y-axis, labeled as AOI (Area of Interest), indicates the proportion of target fixations within a specific time bin, with values ranging from 0 to 1 (see Appendix D).

In these figures, the lines represent mean fixation proportions to the target image over time. The standard error (SE) ribbons around these lines indicate the precision of our estimate. The SE shows how much individual participants' fixation patterns deviate from the mean at each time point. A narrow ribbon indicates that most participants behaved similarly. A wider ribbon suggests more variability between participants.

In these figures, the grey-shaded CPA clusters indicate statistically significant time bins where predictive-looking behavior occurred. Predictive-looking behavior describes when participants look at the target object (e.g., cake) before hearing it because of what they encountered early on in the sentence (e.g., eat). These CPA clusters help us pinpoint precisely when participants began to anticipate the target object based on the linguistic input they had heard, even before the object itself was named in the sentence. By visualizing these clusters, we can identify subtle patterns in participants' eye fixations that might be difficult to discern from numerical data alone.

## Appendix D

### Eye-tracking metrics

Fixation proportions represent whether participants were looking at a specific picture (Area of Interest or AOI) at any given moment. This is initially coded as a binary measure: 1 if the participant was looking at the target picture and 0 if they were not.

For each time bin, typically every 100 ms, we have a 1 or 0 for each participant. We then average these binary values across all participants for that specific time bin. This average gives us a proportion between 0 and 1. For example, if at a given time bin, 7 out of 10 participants were looking at the target picture, the fixation proportion would be 0.7 or 70 %. We repeat this process for each time bin throughout the trial.

We calculated fixation proportions using R, a statistical programming language. The code for these calculations is available in our OSF repository to allow the replication of our results (https://osf.io/6us4d/?view_only=37ab6e17dcc7436b9b21d685e8690c70).

## Appendix E

### ChatGPT prompts used to generate items for Experiment 3

A. The prompt for generating experimental items

I want to write 16 experimental sentences for a self-paced reading experiment to examine syntactic priming effects. All experimental items will contain two NPs (NP1, NP2).

In 8 of the experimental items, the structure of the NP1 should be identical to NP2, but the lexical items should be different. Specifically, NP1 will contain a pre-nominal adjective phrase (e.g., a strong prince), and NP2 will include a different pre-nominal adjective phrase (e.g., a young doctor). In the other 8 experimental items, the structure of NP1 should be different than NP2. Specifically, NP1 will be a relative clause (e.g., a knight that is brave), while NP2 will be a pre-nominal adjective phrase (e.g., a strong prince).

- The lexical items should be frequent and easy to understand for L2 English speakers.
- The lexical items should be different in each sentence
- The length of experimental items should range between 10 and 14 words.

Examples are as follows:

Experimental item conditions:

A: Similar (NP1 = AdjP, NP2 = AdjP) = "A clever lawyer and a greedy businessman made a deal"

B: Different (NP1 = Relative clause, NP2 = Adjp) = "A lawyer who is clever and a greedy businessman made a deal"

Following the above rules, please construct 16 unique sentences: 8 for the similar condition, 8 for the different condition. Please ask clarifying questions if something is not clear.

B. The prompt for generating filler items

I want to create 32 fillers to be used in a self-paced reading task to examine the effects of syntactic priming.

- The sentence fillers should never include non-modified NPs.
- The sentence fillers should include only one main independent clause, without a dependent clause.
- The sentence fillers should include an equal mix of active and passive syntactic structures.
- The lexical items in the sentence fillers should be frequent and easy to understand for L2 English speakers.
- The lexical items in the sentence fillers should not repeated more than 2 times across sentences
- The length of sentence fillers should range between 10 and 14 words.

Please construct 32 filler sentences following the above criteria. Provide the sentences in a table that mentions their structure type.

C. The prompt for generating comprehension questions for all items

Please create short yes/no questions with removed subjects (or replaced by pronouns) for each sentence.

- Half of the yes/no questions for the "similar condition" should be answered "yes", and the other half "no".
- Do the same for the "different condition".
- A question that should be answered "no" should include a new object that was not mentioned in the original sentence.
- In contrast, a question that should be answered "yes" should include the same object that was mentioned in the original sentence.
- Please keep the adjective modifying the objects as they are without a change.

## Footnotes

Appendix A Supplementary data to this article can be found online at https://doi.org/10.1016/j.heliyon.2025.e42083.

## Footnotes

1 For more details about these effects, we refer the reader to the original/related studies involving them, as space constraints prevent a fuller discussion here.

2 All materials for this experiment are available at: https://osf.io/6us4d/?view_only=37ab6e17dcc7436b9b21d685e8690c70.

3 These L1 backgrounds are Italian (n = 5) Spanish (n = 3) Portuguese (n = 3) Hebrew (n = 1) Bulgarian (n = 1) Polish (n = 1) Afrikaans (n = 1) Cantonese (n = 1) Hungarian (n = 1) Russian (n = 1) Dutch (n = 1) and Greek (n = 1).

4 These L1 backgrounds included Portuguese (n = 9) Polish (n = 7) Spanish (n = 6) Italian (n = 3) Greek (n = 2) Russian (n = 1) Hungarian (n = 1) and Czech (n = 1).

5 All materials for this experiment are available at: https://osf.io/6us4d/?view_only=37ab6e17dcc7436b9b21d685e8690c70.

6 The L1 backgrounds included Tagalog (n = 12) Urdu (n = 10) Bengali (n = 3) Thai (n = 3) Filipino (n = 2) Malay (n = 2) Bambara (n = 1) French (n = 1) Fula (n = 1) Indonesian (n = 1) Iranun (n = 1) Mandinka (n = 1) Pashto (n = 1) Persian (n = 1) Tausug (n = 1) Uzbek (n = 1) Wolof (n = 1). Based on an Arabic c-test, we categorized the L2 participants' proficiency levels as intermediate (n = 28), lower (n = 7), and higher (n = 8).

7 All materials for this experiment are available at: https://osf.io/6us4d/?view_only=37ab6e17dcc7436b9b21d685e8690c70.

8 The L1 backgrounds included: Polish (N = 3), Spanish (N = 2), Hungarian (N = 1), Vietnamese (N = 1), Portuguese (N = 1), Hindi (N = 1), and Estonian (N = 1). All L2 participants have completed at least some college education. The participants' mean age was 27 (SD = 6.76 6.76, Range = [20,45]).

9 The L1 backgrounds included: Polish (N = 9), Portuguese (N = 8), Czech (N = 1), Greek (N = 2), Italian (N = 4), Spanish (N = 4), Slovenian (N = 1), Afrikaans (N = 1), and Hungarian (N = 1).
