# Supplementary material for: The acceptability and validity of AI-generated psycholinguistic stimuli
Source: Heliyon. 2025 Jan 17;11(2):e42083. doi: 10.1016/j.heliyon.2025.e42083 (PMC11791230; doi:10.1016/j.heliyon.2025.e42083)
Supplement: Multimedia component 1 [file mmc1.docx]

# Supplementary materials

## Model Structure

### Experiment 1

The R package permutes (Voeten, 2023) was used to develop one CPA model. The model included the independent variable Trial Type (.-.5 baseline, .5 prediction) and random slopes for participants and items. It included data from the verb region (TW1).

Logistic regression mixed effects models were built using lme4 (D. Bates et al., 2015) since the dependent variable Target Fixation is binary. Two models were constructed per region. In both models, the independent variables included Trial Type (.-.5 baseline, .5 prediction), and scaled Time (M = 0, SD = 1).

### Experiment 2

CPA was performed using the R package permutes (Voeten, 2023). Four CPA models were built separately for each pair of Group (L1 speakers, L2 speakers) and Study (Study 1, Study 2). All CPA models included the independent variable Trial Type (baseline = -.5, prediction = .5) and random slopes for participants and items. All models only included data from the verb region (TW1).

Logistic regression mixed effects models were built using lme4 (D. Bates et al., 2015). In the main models, the independent variables included Trial Type (baseline = -.5, prediction = .5), scaled Time (M = 0, SD = 1), Group (L1 speakers = -.5, L2 speakers = .5), and Study (Study 1 = -.5, Study 2 = .5).
